# An agent-based model of prostate Cancer bone metastasis progression and response to Radium223

**DOI:** 10.1186/s12885-020-07084-w

**Published:** 2020-06-29

**Authors:** Stefano Casarin, Eleonora Dondossola

**Affiliations:** 1 Center for Computational Surgery, Houston Methodist Research Institute, Houston, TX USA; 2 Department of Surgery, Houston Methodist Hospital, Houston, TX USA; 3 Houston Methodist Academic Institute, Houston, TX USA; 4 David H. Koch Center for Applied Research of Genitourinary Cancers, The University of Texas, MD Anderson Cancer Center, Houston, TX USA

**Keywords:** In silico model, Radium 223, Prostate cancer bone metastasis, Tumor growth, Therapy response, Therapy optimization

## Abstract

**Background:**

Bone metastasis is the most frequent complication in prostate cancer patients and associated outcome remains fatal. Radium223 (Rad223), a bone targeting radioisotope improves overall survival in patients (3.6 months vs. placebo). However, clinical response is often followed by relapse and disease progression, and associated mechanisms of efficacy and resistance are poorly understood.

Research efforts to overcome this gap require a substantial investment of time and resources. Computational models, integrated with experimental data, can overcome this limitation and drive research in a more effective fashion.

**Methods:**

Accordingly, we developed a predictive agent-based model of prostate cancer bone metastasis progression and response to Rad223 as an agile platform to maximize its efficacy. The driving coefficients were calibrated on ad hoc experimental observations retrieved from intravital microscopy and the outcome further validated, in vivo.

**Results:**

In this work we offered a detailed description of our data-integrated computational infrastructure, tested its accuracy and robustness, quantified the uncertainty of its driving coefficients, and showed the role of tumor size and distance from bone on Rad223 efficacy. In silico tumor growth, which is strongly driven by its mitotic character as identified by sensitivity analysis, matched in vivo trend with 98.3% confidence. Tumor size determined efficacy of Rad223, with larger lesions insensitive to therapy, while medium- and micro-sized tumors displayed up to 5.02 and 152.28-fold size decrease compared to control-treated tumors, respectively. Eradication events occurred in 65 ± 2% of cases in micro-tumors only. In addition, Rad223 lost any therapeutic effect, also on micro-tumors, for distances bigger than 400 μm from the bone interface.

**Conclusions:**

This model has the potential to be further developed to test additional bone targeting agents such as other radiopharmaceuticals or bisphosphonates.

## Background

Prostate Cancer (PCa) is among the most common cancers in American men, second only to skin cancer, and represents 9.9% of all new cases and 7.1% of the total male cancer deaths [1]. Despite a 5-year survival rate close to 100% for localized tumors [2], disease often progresses towards a metastatic stage, with survival at 5 years dropping to ~ 30%. PCa has high propensity to generate bone metastases (84% of patients) [3], which are associated with shortened survival, deteriorated quality of life [4], and resistance to conventional and molecular-targeted therapies [5].

Radium223 (Rad223), a rare earth metal radioisotope, recently emerged as a promising bone-targeting radiation therapy [6] that prolongs overall survival of patients with metastatic prostate cancer by 3.6 months compared to placebo-treated group [7–9]. Rad223 becomes enriched in bone after administration and the low tissue penetrance of alpha-particles (< 100 μm) generates negligible toxicity both systematically and to the bone marrow compared with beta particles-emitting isotopes [8, 10]. However, a promising initial response is often followed by tumor relapse and subsequent progression. Whether Rad223 effects are indirect, based on microenvironmental reprogramming and tumor growth delay, or direct, through cytotoxicity and elimination of tumor cells, remains mostly unsolved [8, 11–13] because of a poor understanding of Rad223 function and underlying mechanisms of action.

This matter is being investigated in vivo. Mouse models of cancer, however, are limited in addressing the multi-parameter complexity of therapy response in a time-cost effective manner (e.g. dose scheduling, number of combinations and observation time). Mathematical models, supported by computational simulations, are powerful tools that can potentially bridge this gap being able to explore an unlimited number of experimental combinations also avoiding time and resource consumption. In silico systems, integrated with biological data, proved effective to gain a deeper knowledge on several diseases’ mechanisms, serve as interrogation tools for clinicians and biologists to test specific hypotheses, and predict the efficacy of putative therapeutic agents and interventional techniques across heterogeneous fields of research [14]. Accordingly, they are suitable to optimally direct preclinical and clinical applications.

Recent in silico models of PCa bone metastasis [15–17] display an accurate cell phenotype and signaling and replicate both pathophysiological events and therapy response on a portion of bone of arbitrary dimensions, approximated as a passive uniform continuum. Although providing an elegant platform to test the therapeutic response to bisphosphonates and TGF-β inhibition [15, 17], they are not optimal to test agents whose action depends on spatial topology (a key aspect of Rad223-based therapies).

In a previous work, we hypothesized that low tissue penetrance affects the efficacy of Rad223 on tumor cells in bone, with maximum effects towards micro-lesions, and developed a predictive in silico model of pre-established PCa osteolytic bone metastasis to test this hypothesis [13]. Since Rad223 effect is distance-dependent [8], we opted for an Agent-Based Model (ABM), driven by cellular automata (CA), where the agents’ (metastatic cells) dynamic and the effect of Rad223 were calibrated from in vivo-derived data obtained from in house murine experiments.

In the proposed work, we provided a detailed description of the modeling techniques implemented, studied the model’s accuracy and robustness, and quantified the uncertainty of its driving coefficients. We re-calibrated our model on a new set of data to prove its flexibility and verified our previous findings [13] on a different dataset. Finally, we showed that tumor location respect to bone interface (the site where Rad223 accumulates) also affects therapeutic efficacy.

## Methods

### In vivo experimental setup

All the animal studies were approved by the Institutional Animal Care and Use Committee of The University of Texas, MD Anderson Cancer Center, which is accredited by the Association for Assessment and Accreditation of Laboratory Animal Care, and performed according to the institutional guidelines for animal care and handling. 8-weeks old athymic nude male mice (20 g) were purchased from the Department of Experimental Radiation Oncology, M.D. Anderson Cancer Center. Mice were housed with a maximum of 5 animals per cage in a state-of-the-art, air-conditioned, with a 12-h light/12-h dark cycle and food ad libitum, specific-pathogen–free animal facility and all procedures were performed in accordance with the NIH Policy on Humane Care and Use of Laboratory Animals. Surgical procedures were performed with mice under general anesthesia (isoflurane), and analgesia was provided at the end of each procedure (buprenorphine, 0.05 mg/kg, one dose immediately before the start of the surgery, a subsequent dose postoperatively within 24 h). Tumor-bearing and control animals were observed daily and examined by a veterinarian 5 days/week for signs of morbidity (e.g. matted fur, weight loss, limited ambulation, and respiratory difficulty). In case of discomfort, the animals were euthanized by isoflurane inhalation followed by cervical dislocation, consistent with the recommendations of the Panel on Euthanasia of the American Veterinary Medical Association. At the end of the study, mice were euthanized as described above.

#### Tumor growth monitoring in the mouse tibia

PC3 human prostate cancer cells (from ATCC, CRL-1435) expressing luciferase were injected in the mouse tibia, as previously described [13]. Mice were anesthetized with isoflurane, a small cut was performed on the internal side of the thigh to expose the tibia, tumor cells (0.25 × 10^6^ for validation of in silico tumor growth, *n* = 8; 0.1, 0.25, 1, 1.5 × 10^6^ for studying the role of tumor dimension on Rad223 response, *n* = 50, as previously described [13]) injected, the wound clipped, and mice provided with analgesia (buprenorphine). Mice were analyzed by macroscopic bioluminescence using an IVIS 200 imaging system (Perkin Elmer, Waltham, MA). They were anesthetized using isoflurane, injected retro-orbitally with 3.75 mg/ml D-Luciferin (Goldbio, St. Louis, MO), and the photon flux emitted by tumor-bearing tibiae was recorded.

#### Intravital imaging studies

**Fig. 1:**
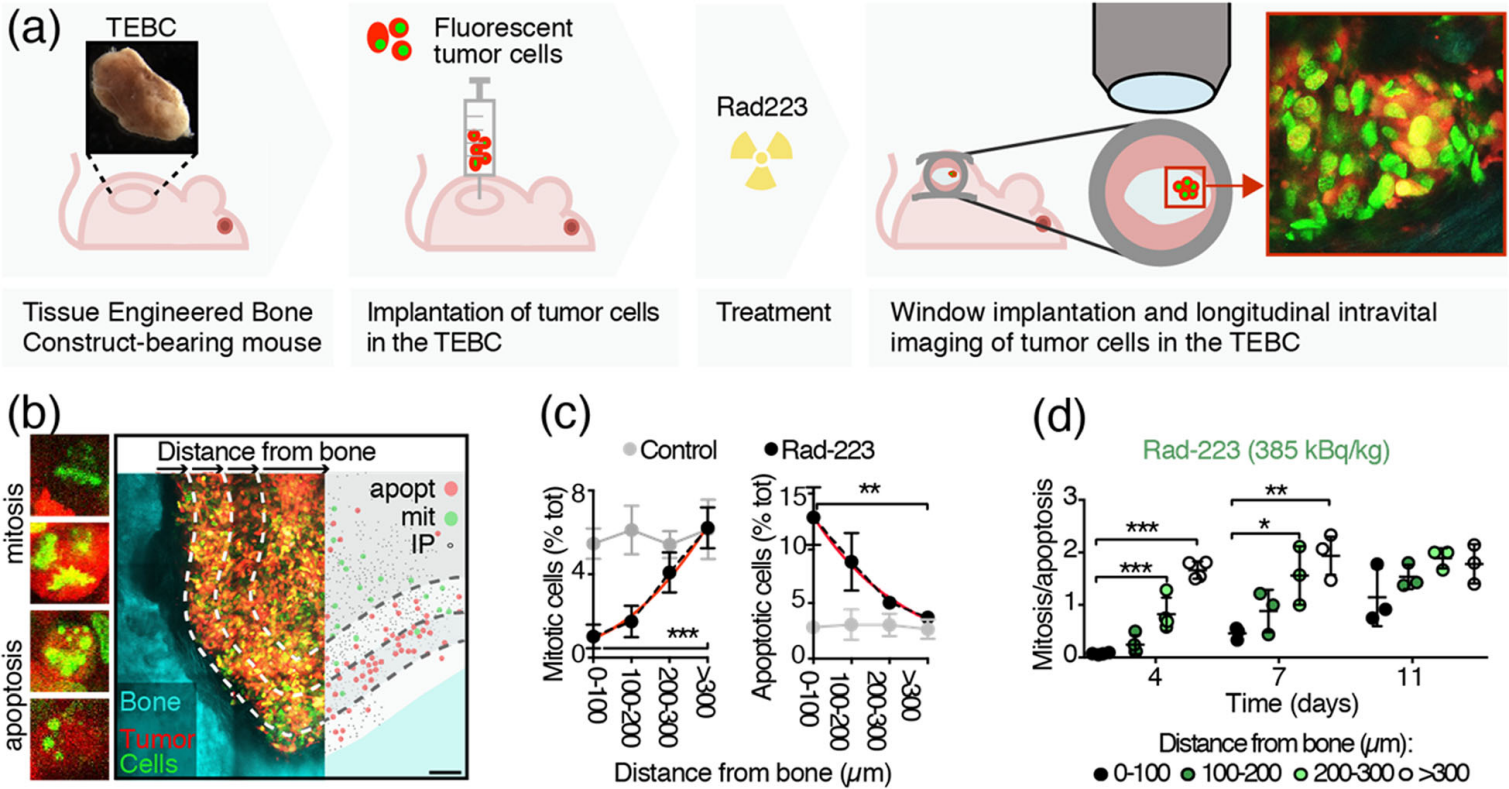
In vivo monitoring of Rad223 response by PCa cell in bone. (**a)** Schematic representation of intravital microscopy workflow: a tissue-engineered bone construct (TEBC) is generated under the skin of the back of a mouse. Upon bone maturation, fluorescent cancer cells are injected in the TEBC, mice treated with Rad223 and monitored by intravital multiphoton microscopy through a window system. (**b**) Exemplificative picture of a tumor lesion in bone, its segmentation every 100 μm and representation of the nuclear status (apopt, apotosis; mit, mitosis; IP, interphase). (**c**) Number of mitotic and apoptotic cells (represented as percent of the total number of cells) at different distances from bone in Rad223-treated and control mice. (**d**) Ratio between mitotic and apoptotic cells at different distances from bone at day 4, 7, and 11 post-Rad-223 treatment. *P* value by one-way ANOVA followed by Tukey’s HSD post-hoc test. Figure reproduced, with permission, from (Dondossola et al., 2019)© Oxford Academic (2019)

These experiments have been performed and described in [13]. Briefly, a tissue-engineered bone construct (TEBC) was generated underneath the skin of the back of immunodeficient mice by implanting a polycaprolactone scaffold embedded with bone morphogenetic protein 7. After its maturation, the TEBC was injected with PC3 dual color cells, which express a green fluorochrome in the nucleus (H2B/eGFP) and a red fluorochrome in the cytoplasm (DsRed2). Then, tumor-bearing mice (*n* = 4) were treated with Rad223 or diluent (0.9% NaCl) and the therapeutic response monitored by intravital multiphoton microscopy through a window system implanted on the back of the mouse (Fig. 1a). The bone was identified by second harmonic generation signal, while cancer cells by green and red fluorescence. PC3 cells induce osteolysis, an imbalanced bone remodeling process skewed towards an increased bone digestion mediated by osteoclasts, which can induce massive bone resorption with formation of osteolytic lesions in calcified tissue [18, 19]. To test which zone of the tumor preferentially responded to Rad223, three-dimensional (3D) reconstructions of the osteolytic lesion were segmented within 100, 200, 300, or greater than 300 μm equidistance from the bone interface, and the number of mitotic and apoptotic nuclei quantified in each zone at day 4, 7, and 11 post-treatment (Fig. 1b). Four days after Rad223 administration, the zone closest to bone (up to 100 μm) displayed an almost quadrupled increase in apoptosis and negligible mitotic frequency compared with farther zones. The amount of apoptotic and mitotic cells in the more distant core reached levels comparable to control-treated tumors, which did not exhibit any zonal distribution. The ratio of mitosis to apoptosis within 0–100 μm near the bone decreased over time to the level of control mice 11 days post-treatment (Fig. 1c, d). These data were implemented with a bottom-up approach to simulate the zonal therapy effects relative to the tumor-bone interface in the ABM described below.

### In silico modeling

The model and its sub-routines were implemented in Matlab® (v. 2018a, MathWorks, Natick, MA, USA). A stochastic character was chosen to replicate the level of noise of in vivo experiments. Accordingly, an ABM driven by CA simulated the growth of a tumor lesion in bone constituted of single cells endowed with individual probabilities of mitosis, apoptosis, or nondividing state, which together determine tumor regression, persistence or progression in response to Rad223.

**Fig. 2:**
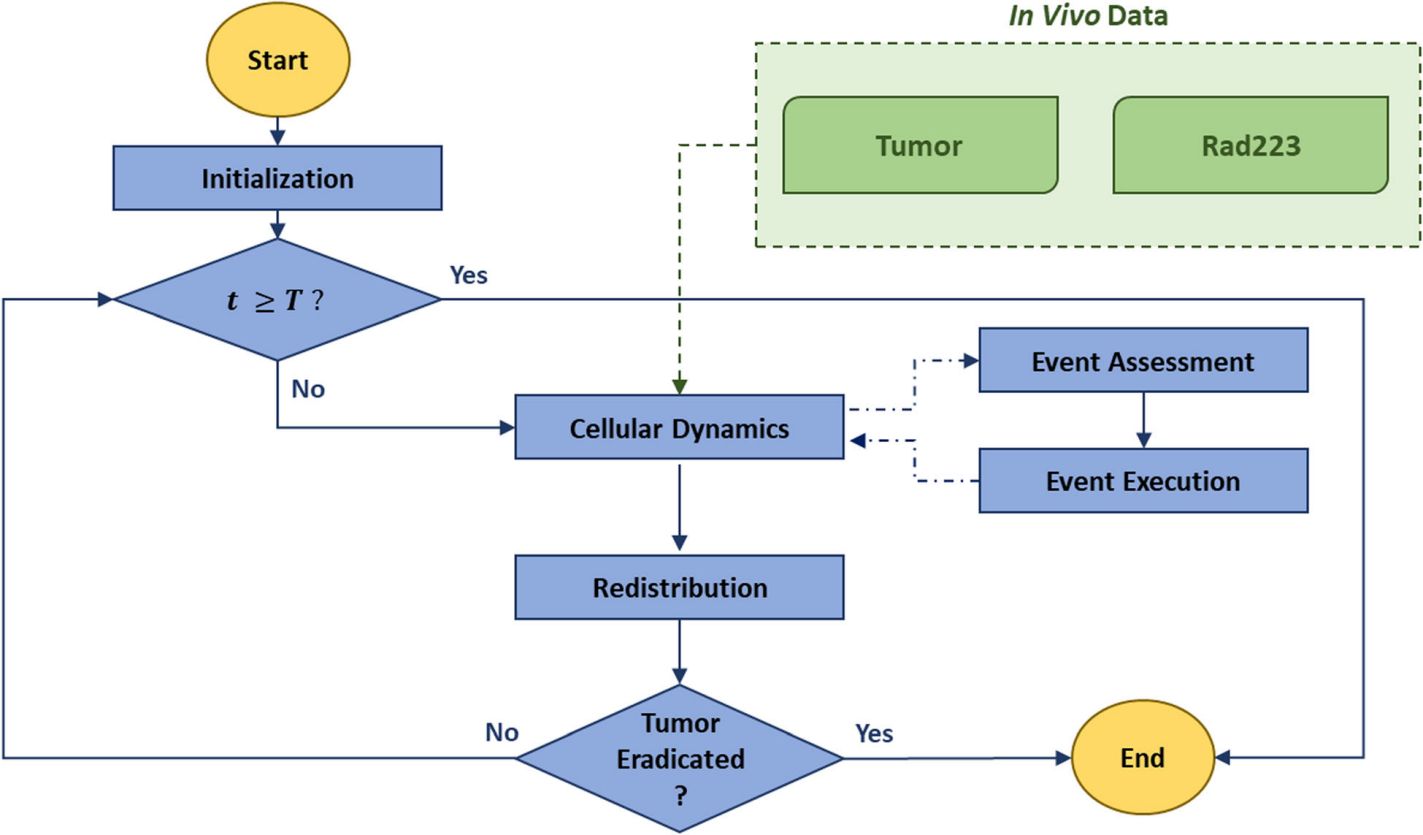
Integrated model flowchart: the yellow circles represent the start and the end of the algorithm, the green portion refers to the integrating in vivo data (Control and Rad), and the blue portion refers to the computational framework, where rectangles correspond to subroutines and rhombi to decisional steps

We simulated a pre-established metastatic lesion of given dimensions and longitudinally tracked tumor size (expressed as number of cells) in Control (untreated) or Rad223 (treated) conditions. The flowchart in Fig. 2 outlines step-by-step the skeleton of the generic simulation (independently on the simulation regimen), which will be described in detail below.

At the beginning of the routine, the model is initialized to replicate the geometry of a metastatic PCa lesion to bone. A first decisional step arrests the simulation if the desired follow-up time has been reached. The algorithm then scans each agent, defines which cell undergoes mitosis, apoptosis, or remains in a quiescent state, and finally executes the outlined cellular events. The probabilities that define the cellular states are specific for the condition simulated (e.g., Control vs. Rad223). To avoid any computational bias leading to the development of a preferred growing direction, a redistribution sub-routine is implemented at the edge of the lesion. The simulation stops if the tumor has been eradicated, otherwise it re-performs the described cycle up to reaching the desired follow-up time, being *T* = 15 *days*.

#### Initialization

The model is implemented on a regular hexagonal grid, which fundamental unit is reported in Fig. S1 (Online Resource 1) [20]. Each agent occupies one site and is surrounded by six neighbors. Regular hexagon is the closest shape to a circle among uniform tessellation opportunities and deriving grids are characterized by the lowest perimeter/area ratio of any other else, which minimizes edge effect. In addition, all neighbors are identical opposed to square tessellation that sees two classes of neighbors: the ones in cardinal direction (shared edges), and in diagonal direction (shared vertexes), which consequently do not have the same distance from the site centroids.

**Fig. 3:**
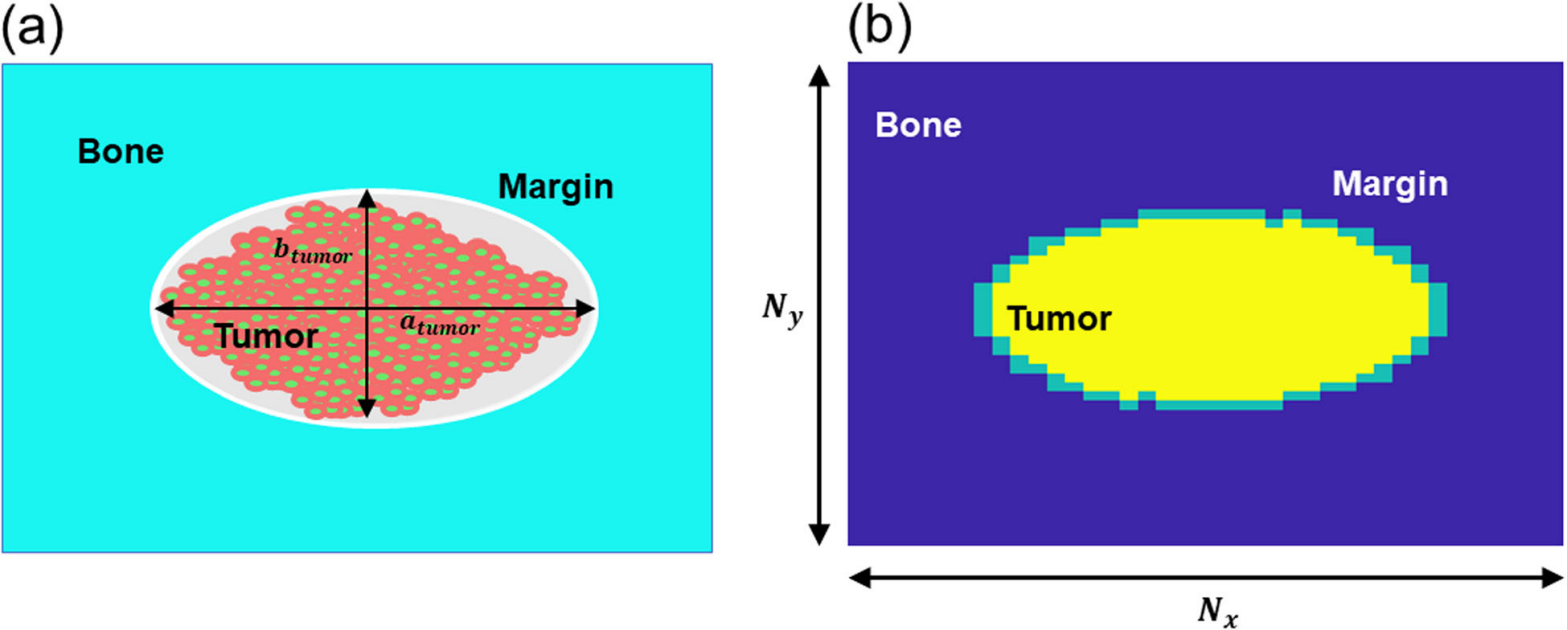
PCa bone metastasis: **(a)** idealized geometry with tumor cells represented by orange circles with green dots, cell-free tumor-bone margin in grey and bone as a uniform continuum in cyan. *a*_*tumor*_ and *b*_*tumor*_ are respectively the horizontal and vertical tumor’s axes. **(b)** computational model initialization with tumor in yellow, margin in green and bone in dark blue. *N*_*x*_ and *N*_*y*_ are the dimensions of the horizontal and vertical grid’s axes

At the beginning of the simulation, the model is initialized to replicate the geometry of an osteolytic bone metastatic lesion that induces bone resorption, represented as a hole in the bone. The overall lesion (Fig. 3a) includes: (i) the tumor, as an ensemble of metastatic PCa cells (orange circles with a green dot, from now on simply referred as cells); (ii) a cell-free tumor-bone margin (in grey) that surrounds the hole; and (iii) a portion of bone (in cyan) as a uniform continuum that is interrupted to form an elliptic hole in correspondence with the tumor. The computational replica approximates the lesion as a 3-domain space (Fig. 3b), where each site of the grid is entirely occupied by its corresponding agent (1 pixel = 1 agent). The tumor is reported in yellow, the margin in green, and the bone in blue. We defined a constant scaling factor (δ) to translate the pixels-based dimension of the ABM into a conventional unit of measure for length (μm). From in house data [13], we observed that a space of 500 μm is occupied by 24 aligned tumor cells, which led to:

1 $$ \delta =\frac{500\mu m}{24}=20.83\ \mu m. $$

Our observation is in line with other published works, which shown an average radius for a single tumor cell of ~ 10 μm [16].

(i) Tumor lesion: The tumor is initialized as an elliptical shape, where *a*_*tumor*_ and *b*_*tumor*_, both expressed in number of pixels/agents are respectively the horizontal and vertical axis, so that the notation [*a*_*tumor*_ x *b*_*tumor*_] uniquely identifies a specific tumor. We tested Rad223 effect on tumors with increasing initial size, specifically [2 × 1], [8 × 7] (micro-tumors), [64 × 53], [128 × 106] (medium-sized tumors), [256 × 213], and [500 × 416] (macro-tumors).
(ii) Cell-free tumor-bone margin: The cell-free tumor-bone margin (simply referred as margin) has initial thickness of 1 pixel and represents the portion of bone virtually occupied by the osteoclasts, the bone resorbing cells. The margin expands with the growth of the tumor lesion, which translates into the digestion of all surrounding bone sites. The margin dynamic is outlined in Fig. S2 (Online Resource 2), where the initial condition (Fig. S2a) represents a generic portion of bone, which is homed by a tumor cell (Fig. S2b) that induced digestion of the closest bone site (Fig. S2c). With the evolution of the tumor, the first step of which is represented in Fig. S2d where the cell has divided, the margin evolves accordingly (Fig. S2e), and the tumor is always separated from the bone by at least one site belonging to the margin. Finally, in case of cell apoptosis, the site left vacant cannot be re-occupied by bone and remains part of the margin (Fig. S2f). As a note, Fig. S2 only represents a generic example for a better reader’s understanding and does not correspond to any specific situation recorded in vivo or in silico.
(iii) Bone: The bone is represented as a unique compartment, in which its cellular components are not made explicit, and occupies the sites of the grids that surround the osteolytic hole where the tumor lesion is accommodated (excluding the cell-free tumor-bone margin). *N*_*x*_ and *N*_*y*_ define respectively the horizontal and vertical dimension of the grid the ABM lays on, which are equal for simplicity (*N*_*x*_ = *N*_*y*_ = *N*). The value of *N*, detailed case-by-case in Table 1, is proportional to the initial size of tumor. The grid needs to be large enough to allow the tumor to grow and the osteolytic lesion in the bone to expand without the mass touching the edge of the grid, causing the simulation arrest. However, choosing by default a very large grid is not an optimal choice since some sub-routines require to scan every site of the grid, dramatically increasing the computational burden if the latter is very large. Thus, we defined specific grids for each tumor size simulated.

**Table 1:** Grid dimensions associated with the initial tumor sizes investigated.

	[a_**tumor**_ x b_**tumor**_]	N
**Small Tumors**	[2x1]	120
	[8x7]	180
**Medium-sized Tumors**	[64x53]	300
	[128x106]	
**Large Tumors**	[256x213]	500
	[500x416]	800

The model is driven by cellular mitosis and apoptosis, as detailed below, which potentially occur with a cadence determined by the regular cellular cycle timespan, assumed to be *T*_*cell*_ = 24 *hours*. This implies that a mitotic or an apoptotic event can occur every 24 h, while, during the intermediate time, the cell remains in interphase state. Accordingly, an internal clock is defined so that a random number ${\hat{t}}_i\in \left[1;{T}_{cell}\right]$ is initially associated to the i-th cell. The clock is updated at every time step of the simulation (*dt* = 1 *hour*) and is reset to zero when ${\hat{t}}_i=24$ to allow the cell to re-start its cycle. The initialization of the clock is randomized to ensure the stochastic nature of the simulation.

#### Cellular dynamics

As shown in Fig. 2, the Cellular Dynamics module is composed by event assessment (where, with a Monte Carlo simulation, the algorithm defines the status of each tumor cell, e.g. mitotic, apoptotic or quiescent), and event execution (where the algorithm modifies the grid occupancy according to the cellular events scheme outlined by the event assessment module).

### Event assessment

Seen the stochastic nature of the model, the mitotic and apoptotic character of each agents was described as a probability density, which driving coefficients were heuristically calibrated from experimental data [13]. For the Control, the probability of mitosis and apoptosis were defined as uniform across the whole lesion, making it every agent to be invested by the same probability density, defined as follows:

2 $$ {p}_{mit}={\alpha}_1=0.4, $$

and

3 $$ {p}_{apop}={\alpha}_2=0.1 $$

**Fig. 4:**
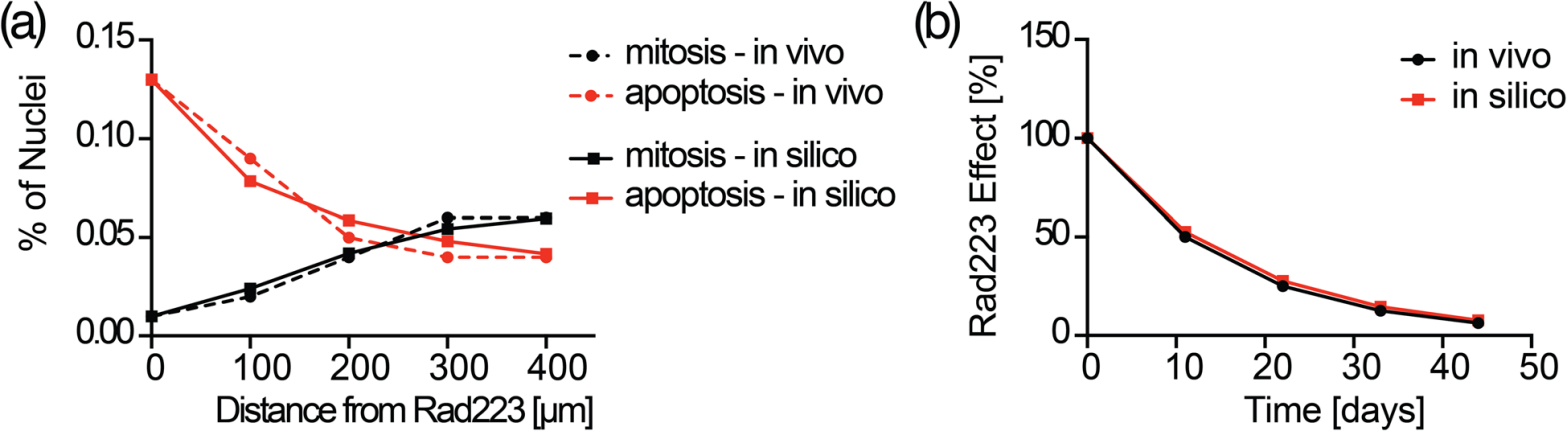
Rad223 coefficients’ calibration: **(a)** distance-dependent effect of Rad223 on mitosis (black dashed – in vivo; black solid – in silico) and apoptosis (red dashed – in vivo; red solid – in silico). **(b)** time-dependent effect’s decay of Rad223 (black solid – in vivo; red solid – in silico)

On the contrary, for Rad223, the bone is turned into a reservoir of this drug. The probability density of each agent depends now both on (i) the distance between site and bone (where Rad223 resides) and (ii) the time, since Rad223 effect decays with a half-life time of 11 days [7]. This modifies Eq. (2) and Eq. (3) in:

4 $$ {p}_{mit}=\frac{\alpha_1}{1+\left[\frac{\alpha_1}{\varphi_{mit}\left({d}_{min}\right)}-1\right]\ast \zeta (t)} $$

5 $$ {p}_{apop}=\frac{\alpha_2}{1-\left[1-\frac{\alpha_2}{\varphi_{apop}\left({d}_{min}\right)}\right]\ast \zeta (t)} $$

where *d*_*min*_ is the minimum distance from the generic tumor agent and the bone, *t* is the time, *φ*_*mit*_(*d*_*min*_) and *φ*_*mit*_(*d*_*min*_) account for the distance-dependent effect of Rad223 respectively on mitosis and apoptosis, and *ζ*(*t*) modulates the time-dependent effect of Rad223, which does not vary between mitosis and apoptosis. From the analysis of the experimental data, a logistic function was the most suitable to replicate the distance-dependent effect of Rad223 on both mitosis and apoptosis. The general form writes:

6 $$ {\varphi}^{\prime }(d)= r\varphi (d)\left(1-\frac{\varphi (d)}{K}\right), $$

where *K* individuates the asymptote, *r* the growth rate, and the initial condition corresponds to the initial value *φ*(0) = *φ*(*d* = 0). We quantitatively calibrated *φ*(*d*) by fitting Eq. (6) onto the experimental data derived for mitosis and apoptosis. To do that, we used a genetic algorithm (pre-built function *ga* from Matlab® Optimization Toolbox) that minimizes the distance between the function *φ*(*d*) parameterized in *K* and *r* and the data. Said distance is defined as the Root Mean Square Deviation (RMSD) between the function and the data and writes

7 $$ RMSD=\sum \limits_{i=1}^N\sqrt{\frac{1}{N}{\left(\varphi (i)-\hat{\varphi}(i)\right)}^2} $$

where *N* is the number of data points collected, $\hat{\varphi}(i)$ the i-th experimental sample, and *φ*(*i*) the i-th value interpolated from Eq. (6). The results of the fittings are reported in Fig. 4a where experimental data are represented with a dashed line and fitted data with a solid one; mitosis-related data are reported in black, while apoptosis-related data are in red. Figure 4a shows how a logistic trend is suitable to fit both the trends accounting for a percentile error < 2% for both cases.

Finally, the decay of Rad223 activity over time was modeled with an exponential function, writing:

8 $$ \zeta (t)={R}_0{e}^{-\frac{t}{\tau }} $$

with *R*_0_ being the initial value, which is also the maximum activity level of Rad223, imposed to be *R*_0_ = 1. The activity decay acts indeed as a modulating mask for Rad223 ranging from 1 to 0 (from 100 to 0% in percentile). In addition, *τ* =  − 0.06 is the decay constant, which value was quantitatively retrieved by fitting Eq. (8) on the correspondent experimental data with same modalities used to fit Eq. (6), i.e. minimization of the RMS via genetic algorithm. The results of the fitting are shown in Fig. 4b which offers a proof of how the time-dependent trend of Rad223 is replicated with negligible error.

Once outlined the densities of probability for both the events in both regimens, driven by a Monte Carlo simulation, the algorithm defines the status of each tumor agent by comparing its event probability with a number randomly generated by the CPU, labeled as *test*. If the agent is in its potential active state (every 24 h), determined by the internal clock status, and the event probability is higher than *test*, than the event occurs. If one condition fails, then the event does not occur, and the agent will be re-evaluated within the next cycle. In summary, the Monte Carlo simulation draws a mask that defines which cells are about to divide, which ones are about to die, and which ones remain in a quiescent state.

### Event execution

Mitosis and apoptosis are executed differently whether the active cell is located at the edge of the tumor or within its body. We individuated four distinct cases which will be described separately:

- Mitosis at the edge
- Mitosis within the body
- Apoptosis at the edge
- Apoptosis within the body

#### Mitosis at the edge

**Fig. 5:**
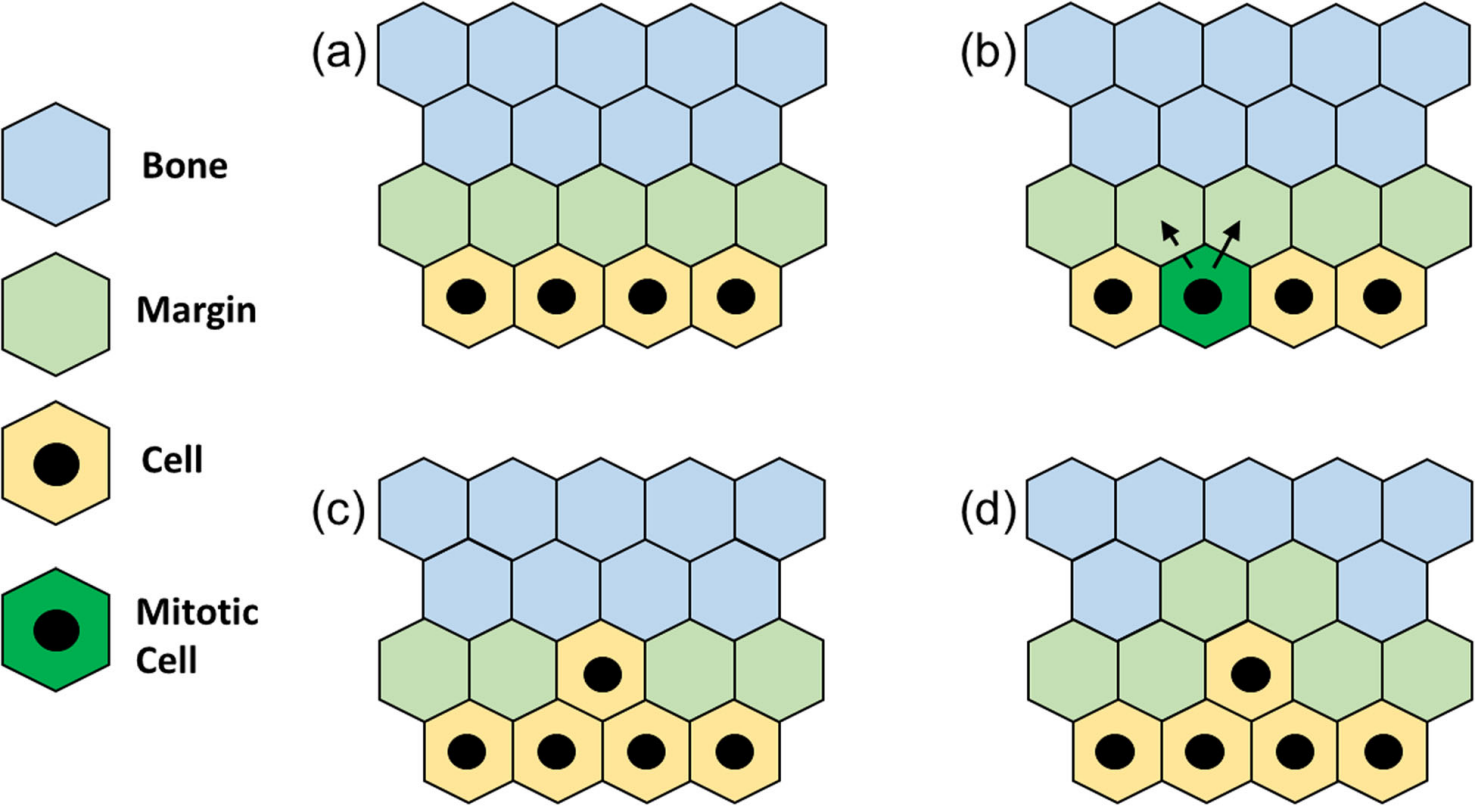
Mitosis at the edge: starting from an arbitrary condition **(a)**, one tumor cell switches to active mitotic status **(b)**, generates a new tumor cell **(c)**, which digests the bone sites around it **(d)**, re-establishing the margin

The steps performed by the algorithm are outlined in Fig. 5. The initial condition (Fig. 5a) sees for simplicity only a portion of the geometry shown in Fig. 3, with tumor cells represented in yellow with a black circle in the middle, the margin in light green and the bone in light blue. Once a cell enters in its mitotic state, represented as a green site in Fig. 5b, it replicates and can deploy the daughter cell in any of the non-tumor adjacent sites, with no preferential target, which is so chosen randomly by the algorithm. In Fig. 5b, the black arrows point to the sites that can potentially receive the daughter with the actual site of reception pointed by a solid arrow, while the other is pointed with a dashed one. Once the reception site has been chosen, it receives the daughter (Fig. 5c), and finally the newly placed cell induces digestion of the remaining bone area around it, re-establishing the 1-pixel minimum thickness of the margin (Fig. 5d).

#### Mitosis within the body

**Fig. 6:**
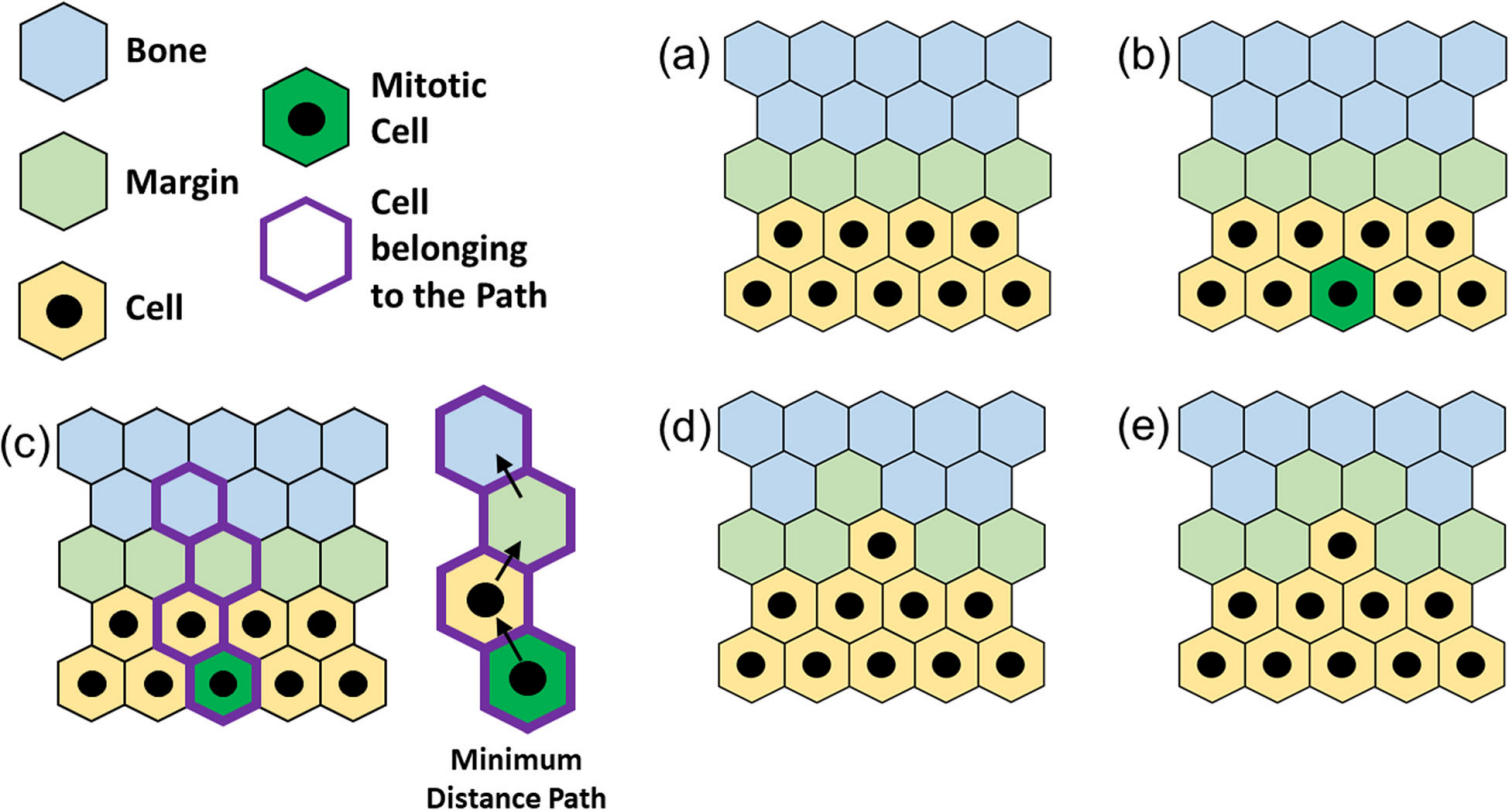
Mitosis within the tumor’s body: starting from an arbitrary condition **(a)**, a tumor cell switches to active mitotic status **(b)**. The algorithm defines the closest bone site to the active cell by drawing a Minimum Distance Path (MDP) **(c)**. Cells on the path are shifted of one position to make room for the newborn cell, which is placed next to the generating one **(d)**. Finally, the newborn cell re-establishes the margin by digesting the surrounding sites **(e)**

The initial condition (Fig. 6a) is alike to the one shown in Fig. 5a, as well as Fig. 6b being alike to Fig. 5b, where the cell in its mitotic state is still identified with a green site. However, while in Fig. 5 the receiving site was at hand, in the current case the structure needs to be re-arranged for the mother cell to have room to place the daughter in one of the six adjacent sites. Figure 6c shows how the algorithm defines a Minimum Distance Path (MDP) between the mother cell and the site belonging to the bone that has the minimum distance from it. The concept underneath this procedure is aligned with the tendency of any biological system to work in the conditions that require the minimum level of energy expenditure among at least two available choices. Following the MDP shown in Fig. 6c, all the sites shift of one entity along said path making room for the daughter cell and expanding the structure towards the outside, where there is now an additional tumor cell (Fig. 6d). Finally, similarly to Fig. 5d, the cell now at the edge induces digestion of the interfacing bone sites and the margin is re-established.

#### Apoptosis at the edge

**Fig. 7:**
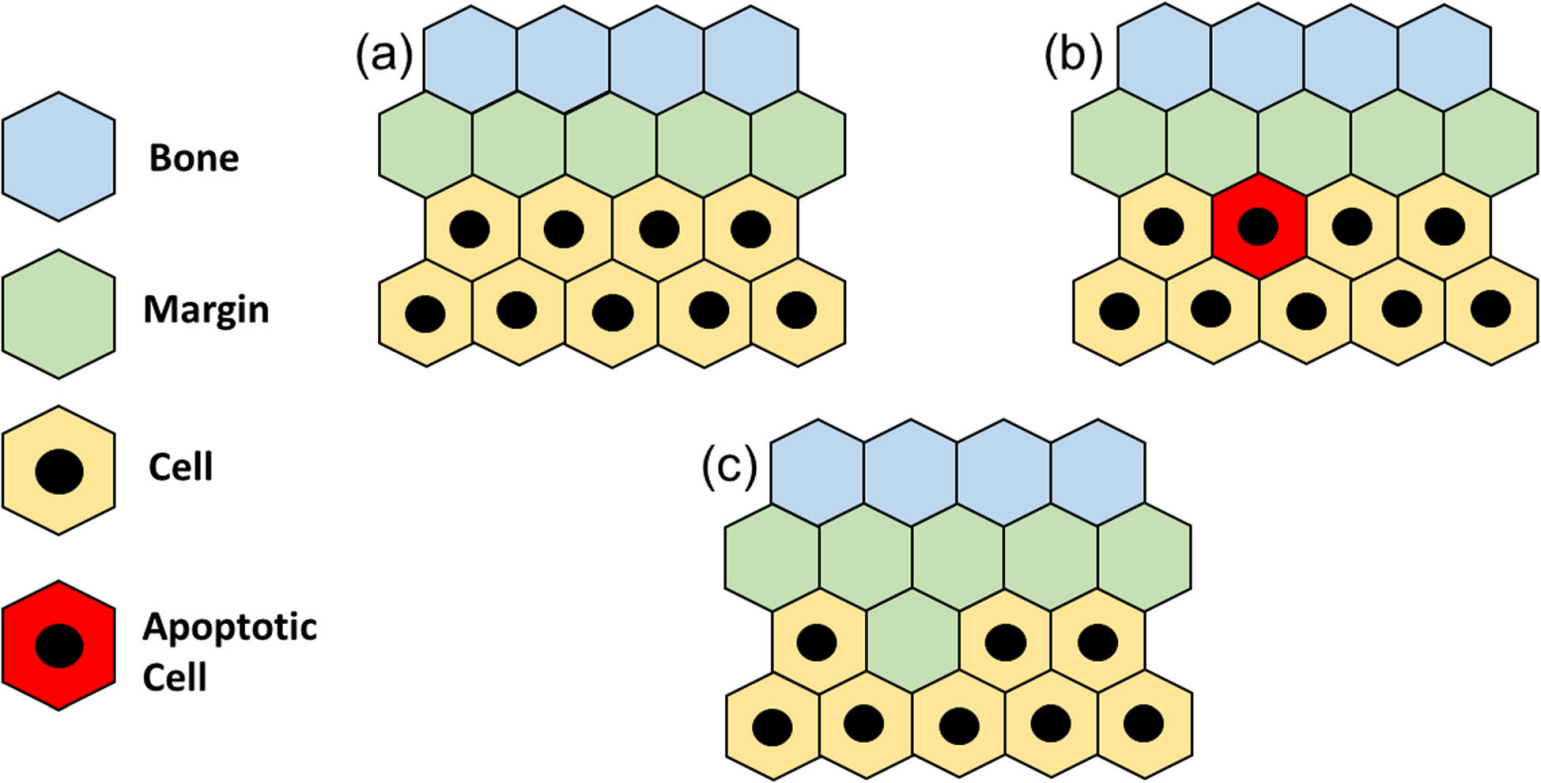
Apoptosis at the edge: starting from an arbitrary condition **(a)**, a tumor cell enters its active apoptotic state **(b)** and vacates the site that becomes part of the margin **(c)**

Being conceptually the easiest case, Fig. 7a shows the initial condition (still alike to the one seen for both mitosis case). As soon as the algorithm has targeted a cell in its apoptotic state, shown as a red site in Fig. 7b, the apoptosis is executed through the cell vacating the site, which becomes now part of the margin (Fig. 7c) and no additional steps are required. As anticipated (Fig. S2f), in case of apoptosis, the now vacant site becomes part of the digested portion and the bone is not allowed to re-grow within it. It is expected that, in presence of tumor regression, the margin grows inward in thickness.

#### Apoptosis within the body

**Fig. 8:**
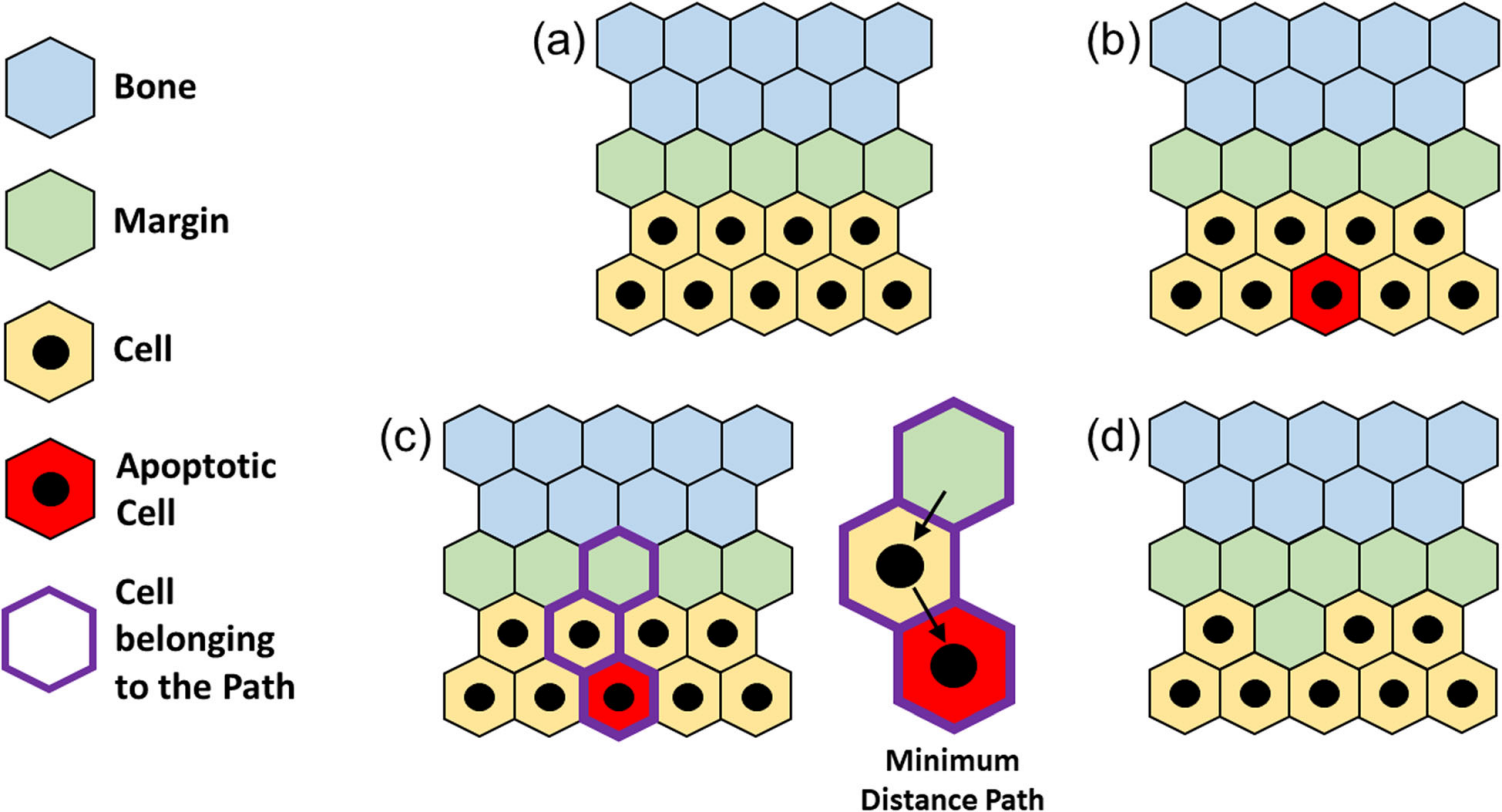
Apoptosis within the tumor’s body: starting from an arbitrary condition **(a)**, a tumor cell enter its apoptotic state **(b)**. The algorithm selects the closest bone site to the active cell by defining a Minimum Distance Path (MDP) **(c)**. The apoptotic cell vacates the site and the cells on the path are shifted of one position inward **(d)**

As seen for the mitosis, Fig. 8a and b represent the same conditions of Fig. 7a and b respectively, where the apoptotic cell is still individuated by a red site. Since the apoptotic cell needs to vacate the occupied site, the structure needs to shrink of one position in order not to leave any hole inside the body of the tumor mass. In this sense, the algorithm operates similarly to what done for the mitosis and individuates a MDP between the apoptotic cell and the closest non-tumor site, which belongs to the margin (Fig. 8c). Consequently, the structure shifts of one position inward along the MDP with result shown in Fig. 8d, where the tumor has retracted of one site and a portion of margin has encroached the tumor mass of one position.

#### Redistribution

**Fig. 9:**
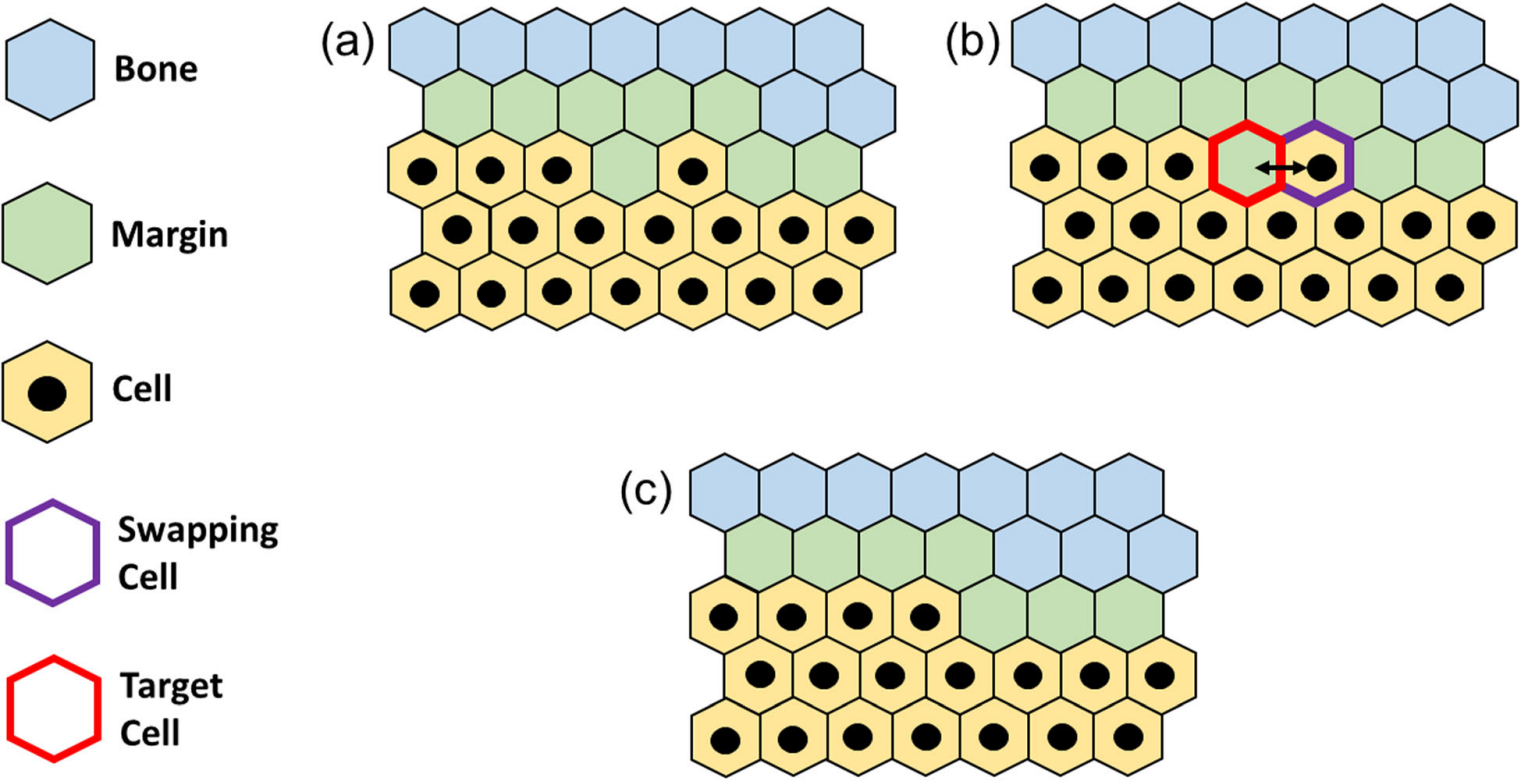
Edge smoothing subroutine: starting from an arbitrary condition **(a)**, a cell with > 3 non-tumor neighbors is labelled as swapping cell and following the dynamics of Fig. S3 a target cell is identified **(b)**. Finally, the two cells are swapped, and the margin re-established **(c)**

To maintain an ellipse-like shape of the tumor for its whole dynamic and consequently avoid the occurrence of any preferential growth direction, we developed an edge smoothing sub-routine by redistributing the tumor cells interfacing with the margin with the rationale shown in Fig. 9.

The origin of a shape disruption was identified by a tumor cell surrounded by more than three non-tumor sites (which can be referred as empty, for simplicity Fig. 9a. A situation alike is highlighted in Fig. 9b, where the bold purple boxed site has a total of four empty neighbors and is individuated as the swapping cell. Once a cell with such characteristic is identified (labeled as cell j in Fig. S3 (Online Resource 3) for the sake of the example), the algorithm individuates a site to be swapped with j to preserve the elliptical shape and guarantee the mass conservation at the same time. Fig. S3, which represents the detail around the swapping cell of Fig. 9b, assists in the understanding of the individuation of the target cell for the swapping, which is done through three sequential steps:

1. The algorithm explores each neighbor of j, labeled as *n*_*i*_ *with i* = 1, …, 6, and keeps track of which neighbors are occupied by tumor and which are not.
2. For each *n*_*i*_, it counts the number of its empty neighbors.
3. The target cell for the swapping is individuated among the empty neighbors, specifically the one that has the least number of empty neighbors itself. In the table of Fig. S3, *n*_4_ and *n*_5_ (shaded in grey) are ruled out not being empty, while *n*_6_ (shaded in red) results being the target cell for the swapping. In case of equal number of empty neighbors between two distinct *n*_*i*_, the target cell will be the one with the lowest distance from the center of the tumor.

Finally, the target cell (boxed in bold red in Fig. 9b) is swapped and the margin is adjusted to keep its thickness equal to 1 pixel in absence of any apoptotic event (Fig. 9c).

#### Analysis of the model

ABMs are intrinsically affected by a certain amount of subjectivity and degrees of freedom [21], which consequently requires an exploration of the model’s behavior. Given the low complexity of the current model, we addressed it by studying the variance of its outputs (i.e. its stability) and how it impacts the model’s design (e.g. number of simulations) and the state of sensitivity to a variation of its driving coefficients.

### Variance of the outputs

The output of the presented ABM was evaluated as the mean of a certain number (*M*) of independent simulations (each of them labeled as *S*_*i*_ *with i* = 1, …, *M*) and each of them virtually corresponding to a single experimental specimen. The choice of *M* inevitably impacts the time needed to run a complete analysis. Table 2 summarizes the average CPU time (minute-approximated) to run a single simulation for each tumor (64-bit single Processor Intel(R) Xeon(R), 2.30GHz, RAM 80.0GB), also differentiated between Control and Rad223 regime, which makes clear how *M* should be chosen by compromising between accuracy and computational feasibility. The optimal choice of *M*, according to Lee et al. [21] should be driven by the pursue of the minimum number of samples that guarantees the stabilization of the coefficient of variation (*C*_*V*_) vs. *M* over a lower asymptote tending to zero, where *C*_*V*_ is defined as

9 $$ {C}_V=\frac{1}{M}\sum \limits_{i=1}^M\frac{\sigma_{S_i}}{\mu }. $$

**Table 2:** CPU running time for a single simulation (Control vs. Rad).

Tumor Size	CPU Time	
	*Control*	*Rad*
[2x1]	1m	< 1m
[8x7]	5m	1m
[64x53]	8m	6m
[128x106]	16m	25m
[256x213]	44m	1h05m
[500x416]	1h32m	4h13m

${\sigma}_{S_i}$ is the standard deviation evaluated for each independent simulation (*S*_*i*_), and *μ* is the mean trend of the *M* simulations. We investigated the percentile value of *C*_*V*_ for each *M* ∈ [10; 100] with a step *dM* = 10. We also hypothesized that the initial tumor dimension influences the choice of *M* and to validate such hypothesis, we conducted the analysis for small and medium-sized lesions. We excluded large tumors from the analysis as we expected a saturation around a stable *M* after a certain dimension. In addition, the required time to run the analysis for large tumors would have been unsustainable without arrangements that fall outside the scope of the current work. Large tumors will be included in the analysis for verification purposes upon optimization of the CPU time required for a single simulation.

**Fig. 10:**
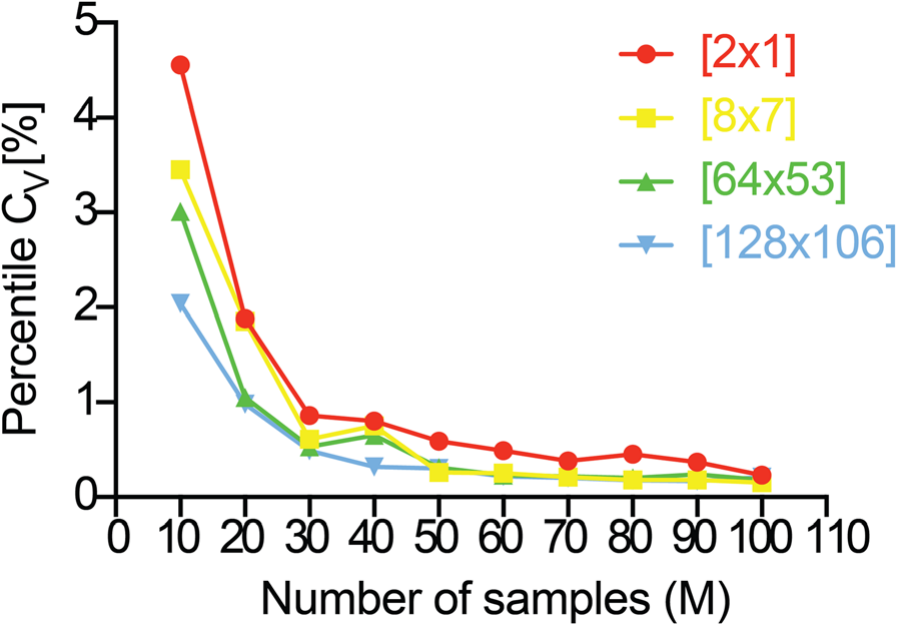
Percentile coefficient of variation (*C*_*V*_) vs. number of independent in silico simulations (*M*) for [2 × 1] (red), [8 × 7] (yellow), [64 × 53] (green), and [128 × 106] (blue) metastasis

Figure 10, showing the *C*_*V*_ vs. *M* plot for each lesion ([2 × 1] red; [8 × 7] yellow; [64 × 53] green; [128 × 106] blue), reports how a reasonable stabilization of *C*_*V*_ is reached with *M* = 70 for [2 × 1], *M* = 50 for [8 × 7] and macro-tumors. First, the results confirmed our hypothesis on the stabilization of *M* beyond a certain initial dimension ([8 × 7]). It is right to assume that *M* does not change from medium-sized to large lesions, setting for the latter *M* = 50. Second, the larger the initial tumor size, the less simulations are required, prospecting a noisier system while dealing with micro-tumors. Third, despite the minimalistic nature of the model (driven by only two coefficients), a high *M* is required*.* For the reasons advanced on the compromise between accuracy and computational feasibility, we chose a less restrictive rationale, by allowing a percentile *C*_*V*_ not higher than 2%. This changed our initial considerations into *M* = 20 for [2 × 1], [8 × 7], and [64 × 53], while for [128 × 106] *M* = 10 already suffices. Finally, the same assumption for macro-tumors is still valid.

### Sensitivity analysis

The output of the ABM is intrinsically affected by the heuristic character of its driving coefficients setup, specifically *α*_1_ and *α*_2_. We performed a sensitivity analysis to (i) quantify the oscillations of the model’s output due to uncertainty on the setup and (ii) to identify which coefficient predominantly drives the model’s dynamic.

For this purpose, a mono-parametric linear sensitivity analysis was carried out on the [8 × 7] lesion. We assumed that the initial size does not affect the output of the analysis. We defined {*α*_1_, *α*_2_} as the vector of coefficients under investigation and for each of them a range (*R*_*i*_ with *i* = 1, 2) of perturbation was defined in the −/+ 50% of variation neighborhood of the chosen value such as

10 $$ {R}_i=\left[{\alpha}_i-0.5\ast {\alpha}_i;{\alpha}_i+0.5\ast {\alpha}_i\right] $$

The range defined with [10] was discretized with a 10% of *α*_*i*_ step, so that a vector of *α*_*i*_ values (${\overrightarrow{R}}_i$ with *i* = 1, 2) was defined with following Eq. (11) and its values outlined distinctly for *α*_1_ and *α*_2_ in Table 3:

11 $$ {\overrightarrow{R}}_i=\left[{\alpha}_i-0.5\ast {\alpha}_i,{\alpha}_i-0.4\ast {\alpha}_i,\dots, {\alpha}_i,\dots, {\alpha}_i+0.4\ast {\alpha}_i,{\alpha}_i+0.5\ast {\alpha}_i\right]. $$

**Table 3:** Mono-parametric sensitivity analysis perturbation vectors.

**α**_**1**_	0.2	0.24	0.28	0.32	0.36	0.4	0.44	0.48	0.52	0.56	0.6
**α**_**2**_	0.05	0.06	0.07	0.08	0.09	0.1	0.11	0.12	0.13	0.14	0.15

One coefficient at the time is varied while keeping the other one at its default value. For each combination of coefficients, the ABM was run *M* = 20 times independently (see the methodology outlined above on the choice of *M*) and the mean value of the final tumor’s size was recorded and normalized on the output of the simulation performed with *α*_1_ = 0.4 and *α*_2_ = 0.1.

## Results

### Model validation

To validate the output of the ABM, we compared in silico and in vivo tumor growth curves at the baseline.

**Fig. 11:**
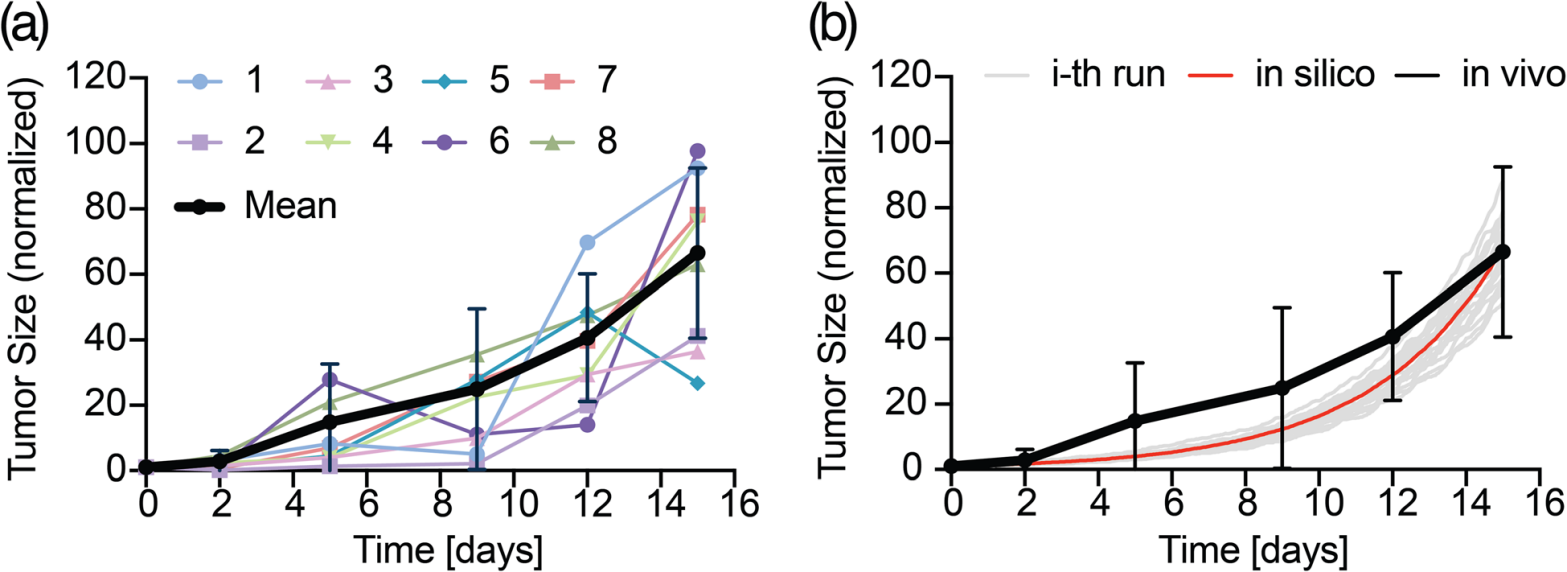
Model validation: **(a)** growth of 8 PC3 tumors (colored lines) in vivo over time and mean temporal trend with standard deviation (black bold). **(b)** in vivo data with standard deviation (black bold) are compared to in silico model output (i-th independent run – light grey; average trend – red bold)

The growth of 8 PC3 tumors (0.25 × 10^6^ cells/tibia), in vivo, was tracked over time and their trend is reported in Fig. 11a. The average growth (black bold line in Fig. 11a along with its standard deviation) was used as reference for model validation.

Since mouse tumors were analyzed at specific time points (0, 2, 5, 9, 12, and 15 days), while the model is investigated with a time step of 1 h, we sampled *x*_*M*_(*t*) in correspondence of said time points. Finally, to be comparable, both discrete trends were normalized on their initial value (corresponding to the initial tumor size). To quantitatively evaluate the distance between our model and preclinical reality, we calculated the Normalized Root Mean Square Error (NRMSE) that writes:

12 $$ NRMSE=\frac{1}{\overline{x_M(t)}}\ast \sqrt{\frac{\sum \limits_{r=1}^L{\left({x}_M^r-{x}_D^r\right)}^2}{L},} $$

where L = 6 is the number of experimental time points.

The temporal dynamics of a [8 × 7] in silico tumor (*x*_*M*_(*t*)) was obtained as the average of *M* independent simulations such as ${x}_M(t)=\frac{\sum \limits_{j=1}^M{x}_M^j(t)}{M}$ .

Figure 11b shows in black the in vivo trend (corresponding to the black bold curve in Fig. 11a) along with its standard deviation, and in red the in silico trend along with its *M* independent simulations’ dynamics reported in light grey. We obtained a high level of confidence with a percentile NRMSE = 1.7% and with the in silico trend fully matching the in vivo within its standard deviation range. For further simulations, we assumed that the value of the calibrated coefficients does not change for lesions of different initial size, so *α*_1_ = 0.4 and *α*_2_ = 0.1 were maintained constant without having to re-calibrate the model for each tumor size.

### Sensitivity analysis

**Fig. 12:**
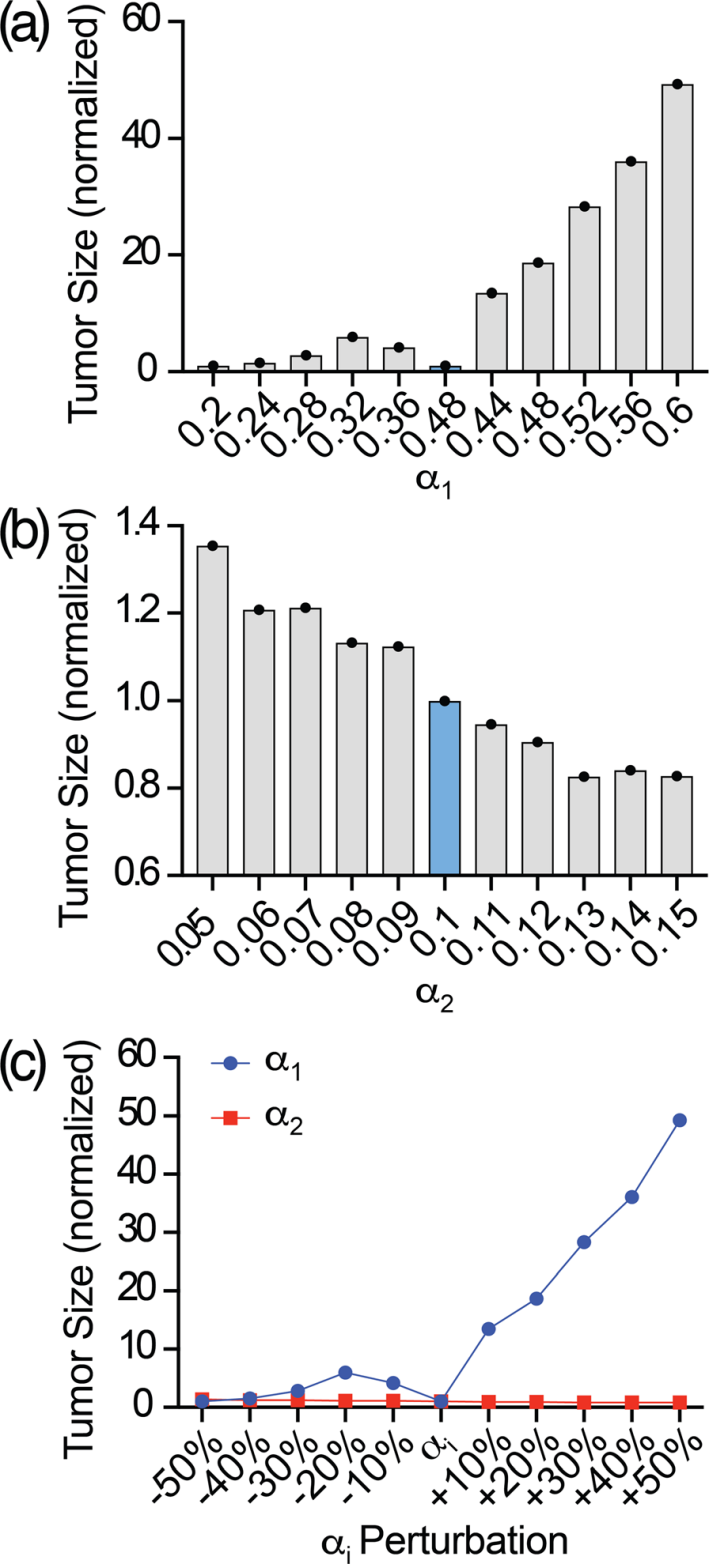
Sensitivity analysis: tumor size variation (normalized) over *α*_1_**(a)** and *α*_2_**(b)** perturbation, with trends compared over the same space scale **(c)**

Figure 12 shows separate results for *α*_1_ (Fig. 12a) and *α*_2_ (Fig. 12b), along with a comparison between the two trends on the same scale (Fig. 12c). Not surprisingly, the model is almost uniquely driven by its mitotic character, the oscillations of which strongly affect the output of the model. An increase of *α*_1_ of 10% from its baseline generates a final tumor size 13 times bigger. On the contrary, the current model is almost insensitive to apoptosis, for which the normalized variation from the baseline does not exceed 0.4 unit. Seen the limited number of coefficients, a mono-parametric analysis is sufficient for the current analysis.

### In silico predictions

**Fig. 13:**
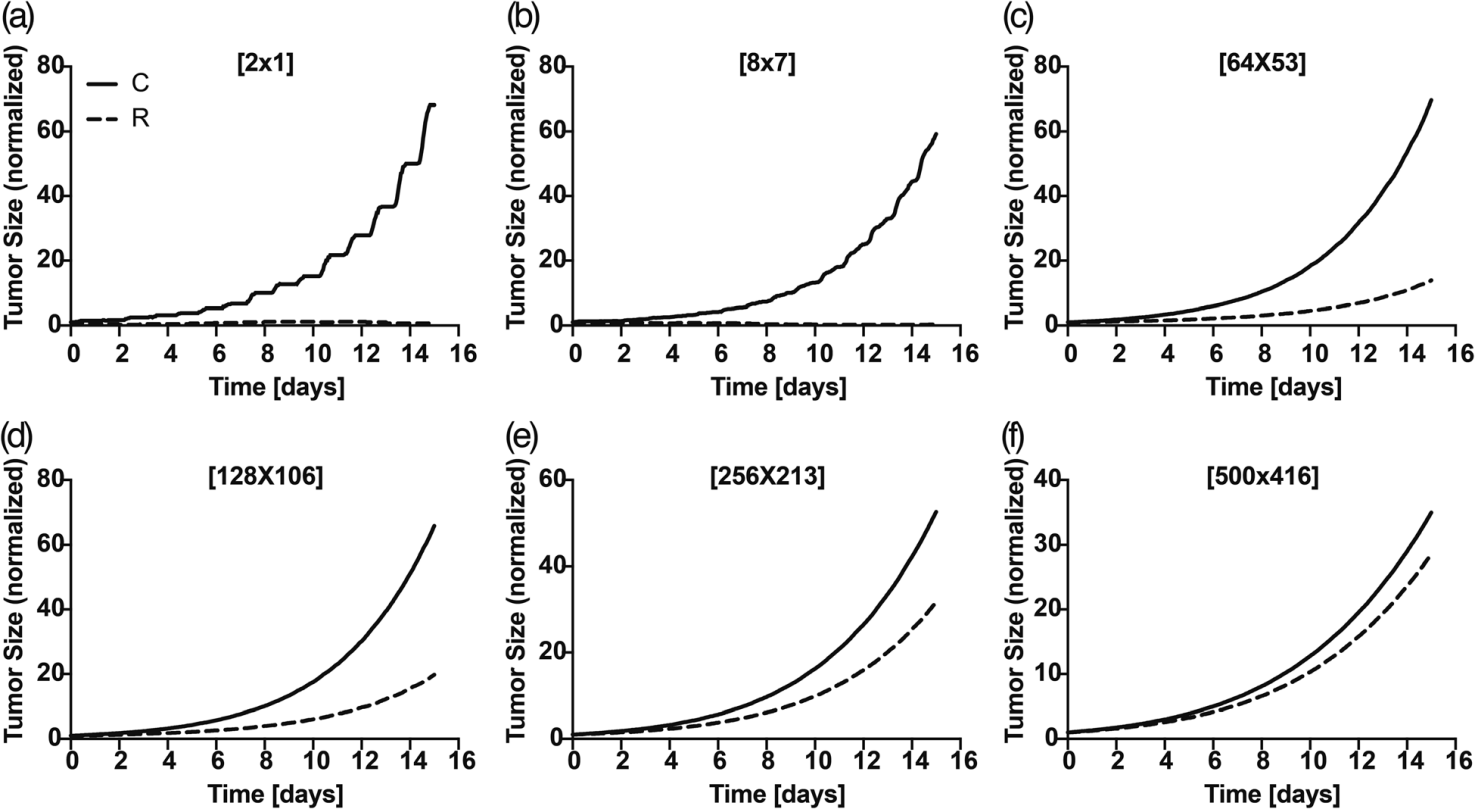
Control (solid line) compared to Rad (dashed line) temporal dynamics in silico for [2 × 1] **(a)**, [8 × 7] **(b)**, [64 × 53] **(c)**, [128 × 106] **(d)**, [256 × 213] **(e)**, [500 × 416] **(f)**

**Fig. 14:**
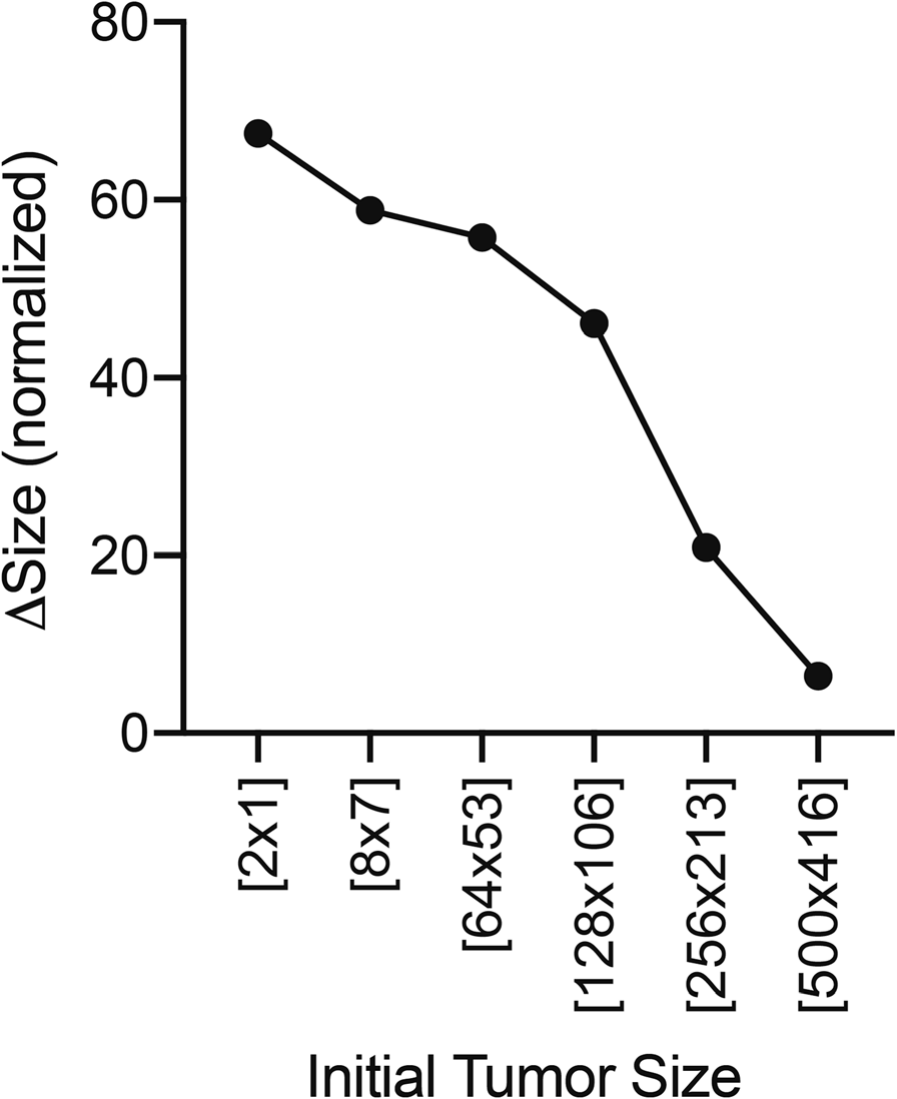
Normalized tumor size difference (Control vs. Rad in silico) at day 15 over initial tumor size

Post validation, we challenged the ABM to test the role of tumor’s size and location. In silico experimental setup was performed by following the rationale detailed in Section 2. For each of the initial tumor size listed in Section 2.2.1, we compared the normalized output in Control (*x*_*M*, *C*_(*t*)) and Rad223 (*x*_*M*, *R*_(*t*)) regimens (Fig. 13) and quantified the difference recorded on the last day of follow-up (day 15). Additionally, for small and medium-sized tumors, we evaluated the percentage of tumor eradication, while we did not expect any eradication event for large tumors. For this analysis, we incremented the number of independent simulations to 1000 for a better robustness of the computational samples. The analysis confirmed our hypothesis that the tumor’s size drives the long-term efficacy of Rad223. From Fig. 13 (qualitative temporal dynamics comparison) and Fig. 14 (quantification of the normalized tumor size difference at day 15), the gap between final untreated and treated lesion size becomes progressively smaller when increasing tumor’s size. Micro-lesions ([2 × 1] and [8 × 7]) show a normalized difference of 67.50 and 58.83 units respectively, corresponding to a 152.29 and 102.25-fold size decrease compared to control-treated tumors. Medium-sized lesions ([64 × 53] and [128 × 106]) display a normalized difference of 55.78 and 46.11 units respectively, which correlate to a 5.02 and 3.33-fold decrease. The trends almost overlap in the largest lesions studied ([256 × 213] and [500 × 416] in Fig. 13f) with a normalized difference of 20.89 and 6.42 units, and a 1.66 and 1.23-fold decrease, respectively. Finally, we only identified eradication events for the smallest lesion ([2 × 1], Fig. 13a), with a percentage of 65 ± 2% over 1000 independent simulations. Thus, the in silico analysis predicts that Rad223-based therapy is most suitable to treat micro-tumors.

**Fig. 15:**
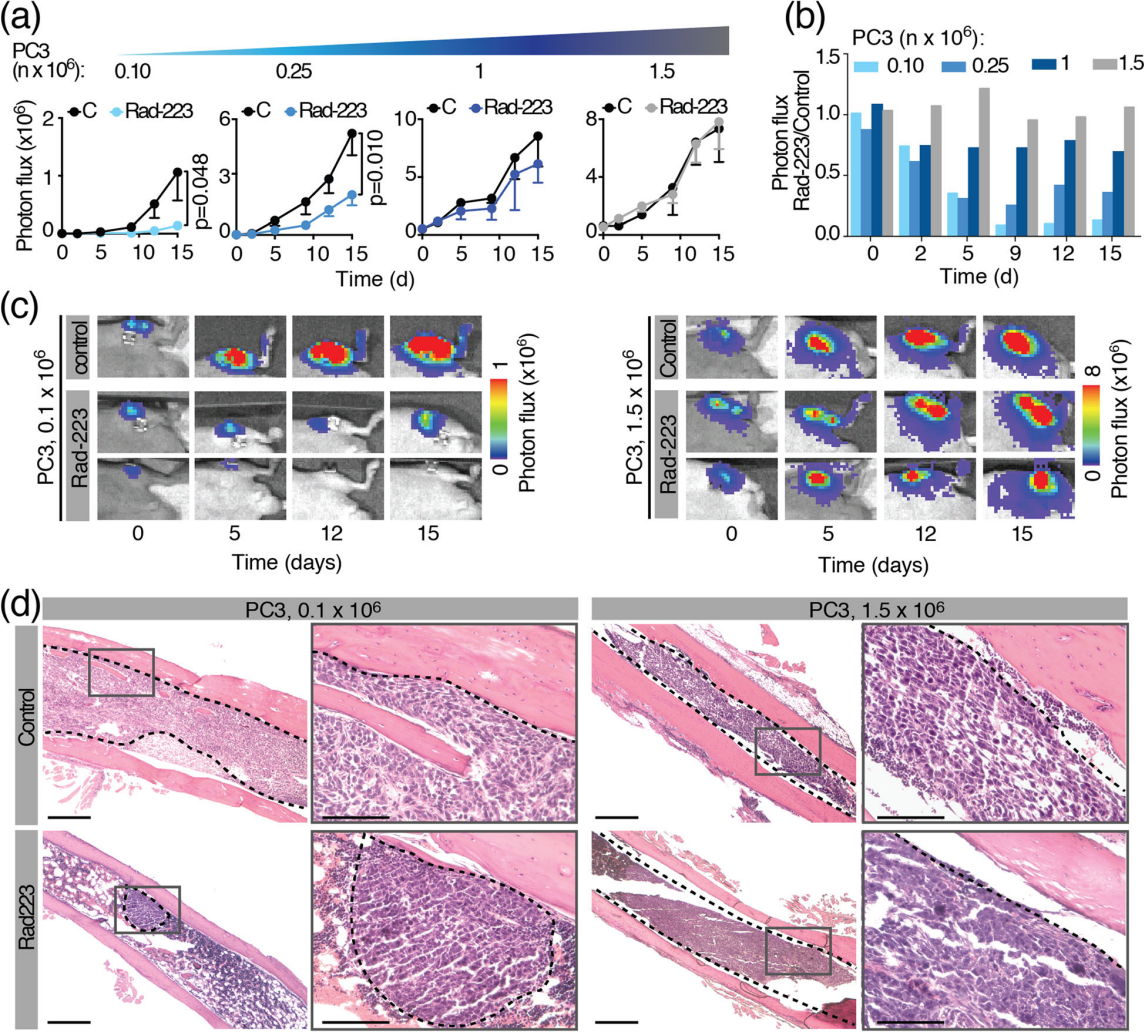
Tumor size-dependent growth control of PCa tumors in bone treated with Rad223. **(a)** Bioluminescence detection of PCa cells (PC3) over time. Mice were implanted with different doses of cancer cells in the tibia and administered with Rad223 or physiological solution (control). **(b)** Time-dependent therapy response expressed as the ratio of the mean bioluminescence of Rad223-treated and control groups over time **(c)** Example images with representative tibiae implanted with 0.10 × 10^6^ and 1.5 × 10^6^ tumor cells are shown. **(d)** End point histology of micro- or macro-tumors after treatment with Rad-223 (day 15). Dashed lines, tumor. Bar = 500 and 100 μm. *P* value by Student *t* test, unpaired, two-sided. Figure reproduced, with permission, from (Dondossola et al., 2019)© Oxford Academic (2019)

These results were further confirmed in vivo, as previously published (Fig. 15 [13];). Mice were implanted with an increasing number of luciferase-expressing PC3 tumor cells in bone (0.1, 0.25, 1, 1.5 × 10^6^ cells/tibia), treated with Rad223 and the response longitudinally recorded. Rad223 significantly reduced the growth of lesions generated with a lower amount of tumor cells (up to 0.25 × 10^6^), whereas tumors beyond 1 × 10^6^ cells kept on growing at a similar extent of Control-treated mice (Fig. 15a-c). These results were further confirmed by histological analysis, showing small nodular lesions in micro-tumors treated with Rad223, and almost total bone marrow replacement in control tumors or Rad223-treated macro-tumors (Fig. 15d).

Then, we broke up the incidence of size and location on the therapeutic efficacy with a two-faced analysis.

**Fig. 16:**
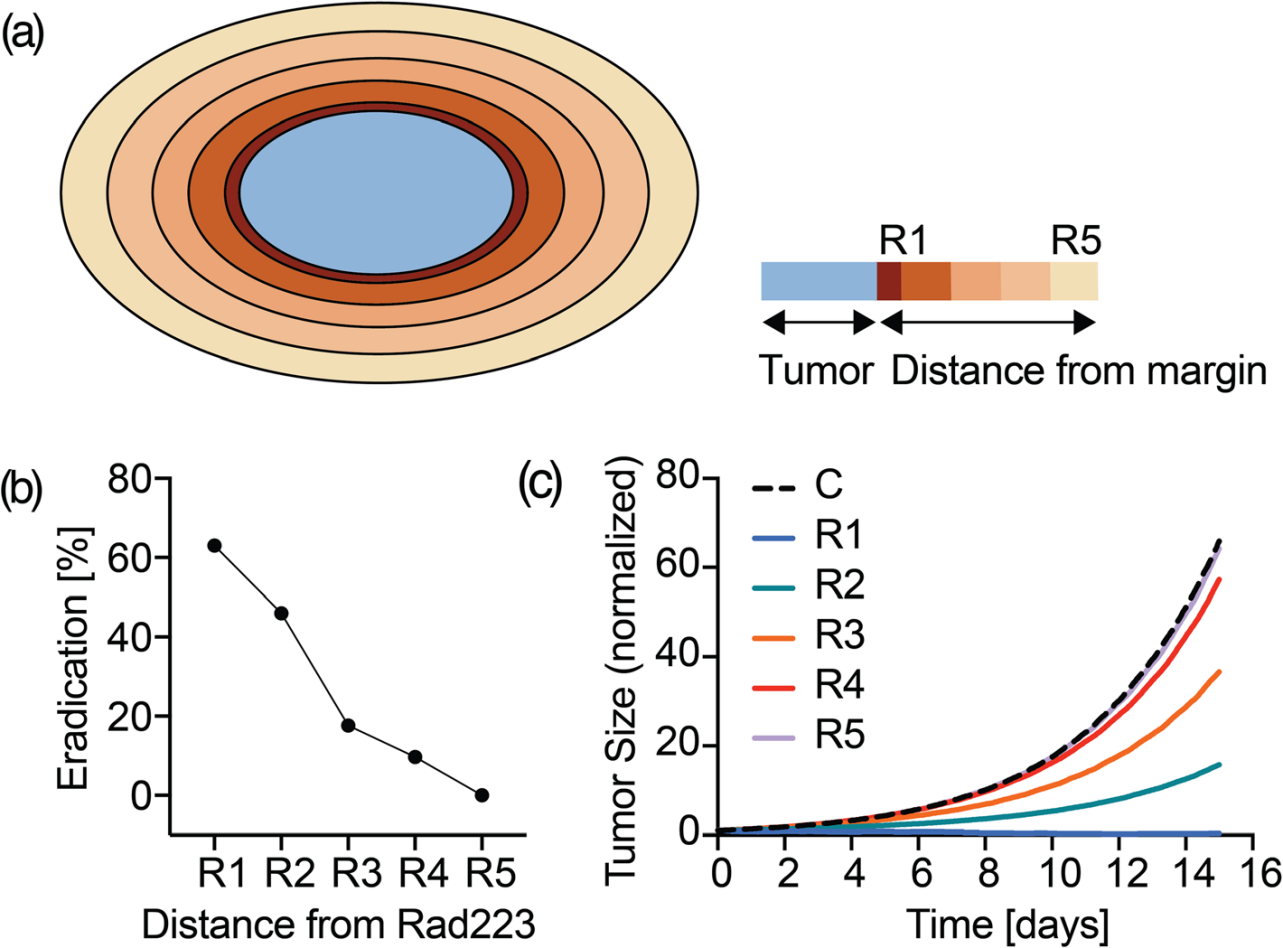
Tumor (in blue) with increasing distance from bone, from dark orange (R1–1 pixel) to light orange (R5–20 pixels) **(a).** Effect of increasing distance from bone evaluated in terms of [2 × 1] tumor eradication events percentage **(b)** and effect of treatment on [8 × 7] tumor at day 15 **(c)**

First, we tested the response of [2 × 1] lesion in terms of eradication for increasing distances (in microns) from Rad223: d < 100 (simulation labeled as R1), 100 ≤ d < 200 (R2), 200 ≤ d < 300 (R3), 300 ≤ d < 400 (R4), and d ≥ 400 (R5), expecting the percentage of eradication to decrease while increasing the distance. Specifically, we investigated a distance of 1, 5, 10, 15 and 20 pixels (Fig. 16a).

Second, we repeated the same analysis reported in Fig. 13b for the [8 × 7] lesion by additionally investigating the effect of an increasing distance from Rad223, progressively augmenting the cell-free tumor-bone margin thickness ranging from simulation R1 to R5. To confirm our speculation, we expect the gap between Control (C) and Rad223 (Ri, with i = 1,…5) to get smaller while increasing the distance from the margin.

From Fig. 16b, the [2 × 1] percentage of eradication decreases as the distance from Rad223 increases. Furthermore, Rad223 loses any therapeutic effect beyond 400 μm, where the chances of eradication are completely abated. As additional evidence, Fig. 16c shows how the benefit of Rad223, already appreciated with Fig. 13b and here re-represented with R1 curve, are progressively nullified as the distance increases. Ultimately, R5 overlaps the Control and shows a normalized tumor size difference of just 1.69 units compared to the original 58.83 (R1). On the base of these evidences, we can speculate that the therapeutic effect of Rad223 is maximum near the bone interface.

In summary, the in silico analysis predicts that cytotoxicity induced by Rad223 targets micro-tumors located near the bone interface, being the site of Rad223 accumulation, with particular efficacy.

## Discussion

By integrating computational techniques and experimental data, we developed a predictive in silico model of pre-established PCa bone metastasis growth and response to Rad223. This system was able to identify micro-lesions close to bone interface as the best target for Rad223-based therapies in terms of regression/eradication, suggesting that patients at initial stages of metastatic progression would benefit more of this treatment. This model has been currently applied to investigate local topological determinants of response upon single administration of Rad223 within 15 days of tumor evolution. As future perspective, such in silico predictions can be applied to simulate longer experimental time, to be matched with survival studies, and different schedules of treatment, including repeated injections and therapy withdrawal.

Besides Rad223, such integrated system represents a suitable tool to pre-test therapeutic approaches that affect bone remodeling, such as other radiopharmaceuticals or bisphosphonates. As a limitation, however, lack of stromal cell detailing prevents testing molecular agents that modulate the biology of specific bone cells (e.g., osteoblasts, osteoclasts), such as kinase or RANKL inhibitors [22, 23].

Thus, a higher model complexity could be implemented, which implies a higher number of independent coefficients and an increased model uncertainty. To cope with it, it will be crucial to clearly differentiate the leading coefficients from the negligible ones, by applying a modular approach, as previously published [24]. With an escalating complexity, we will assist to an increased computational burden that will translate in a dilation of the time needed for a single simulation. To shorten the required computational time, the Matlab® code will be translated in an executable C-code (via Matlab® “coder” Toolbox) and parallel computing techniques will be applied as previously reported [25].

## Conclusions

Clinical response to Rad223 administration is often characterized by relapse and disease progression. This failure is to be ascribed to an insufficient understanding of mechanisms of therapy response and resistance that, due to the multiscale nature of the disease, should be investigated by a conspicuous number of preclinical experiments, implying extended time and vast resource consumption. With this work, we proposed a computational model, integrated with experimental evidences, to overcome this limitation and drive the research in a more effective fashion.

## Supplementary information

**Additional file 1 Fig. S1** Agent-based model fundamental unit (Garbey et al., 2015). **Fig. S2** Cell-free tumor-bone margin dynamics: an arbitrary portion of bone **(a)** is homed by a tumor cell **(b)**, which digests the surrounding neighbors **(c)**. In case of cell mitosis **(d)**, the newborn cell digests the surrounding bone cells **(e)**. In case of apoptosis **(f)**, the cell vacates the site that remains part of the margin. **Fig. S3** Identification of the target cell for edge smoothing subroutine.

## Data Availability

The authors declare that all relevant data supporting the findings of this study are available within the paper and from corresponding author upon reasonable request. Computational code will be provided from corresponding author upon reasonable request.

## Abbreviations

PCa: Prostate Cancer
Rad223: Radium223
ABM: Agent-Based Model
CA: Cellular Automata
TEBC: Tissue-Engineered Bone Construct
RMSD: Root Mean Square Deviation
MDP: Minimum Distance Path
NRMSE: Normalized Root Mean Square Error
