# Supplementary figures and images for: An agent-based model of prostate Cancer bone metastasis progression and response to Radium223
Source: BMC Cancer. 2020 Jun 29;20:605. doi: 10.1186/s12885-020-07084-w (PMC7325060; doi:10.1186/s12885-020-07084-w)

## Supplementary Figure 1

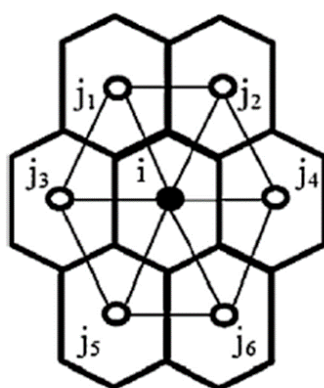

Supplementary Figure 2

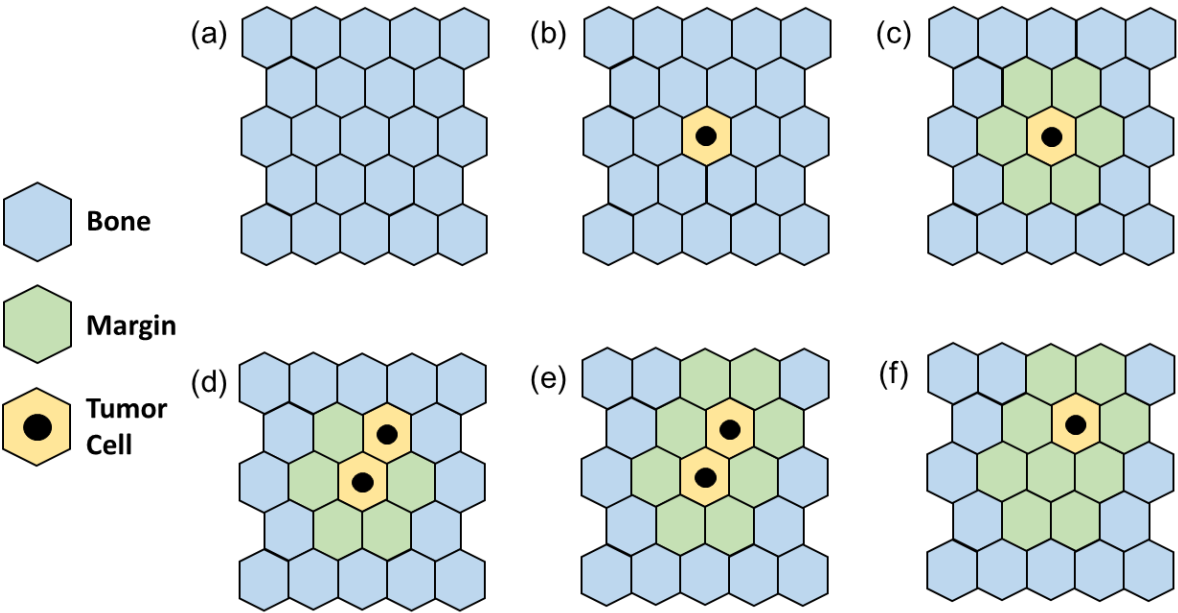

Supplementary Figure 3

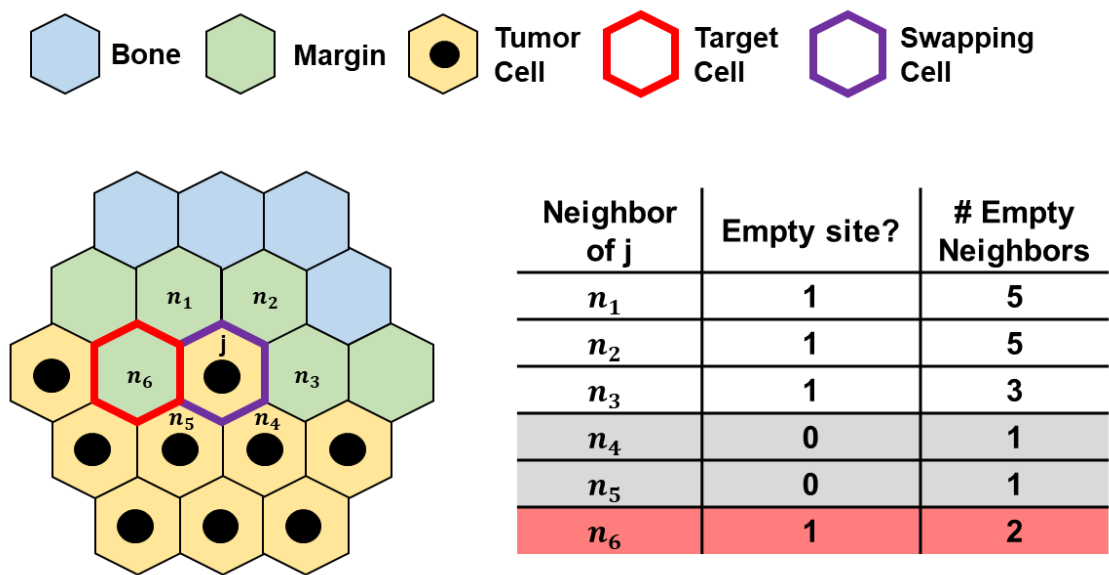

Supplement: Supplementary file 1 — Additional file 1 Fig. S1 Agent-based model fundamental unit (Garbey et al., 2015). Fig. S2 Cell-free tumor-bone margin dynamics: an arbitrary portion of bone (a) is homed by a tumor cell (b), which digests the surrounding neighbors (c). In case of cell mitosis (d), the newborn cell digests the surrounding bone cells (e). In case of apoptosis (f), the cell vacates the site that remains part of the margin. Fig. S3 Identification of the target cell for edge smoothing subroutine. [file 12885_2020_7084_MOESM1_ESM.pdf]
